# Adaptive Combination of *P*-Values for Family-Based Association Testing with Sequence Data

**DOI:** 10.1371/journal.pone.0115971

**Published:** 2014-12-26

**Authors:** Wan-Yu Lin

**Affiliations:** Institute of Epidemiology and Preventive Medicine, College of Public Health, National Taiwan University, Taipei, Taiwan; University of North Carolina, United States of America

## Abstract

Family-based study design will play a key role in identifying rare causal variants, because rare causal variants can be enriched in families with multiple affected subjects. Furthermore, different from population-based studies, family studies are robust to bias induced by population substructure. It is well known that rare causal variants are difficult to detect from single-locus tests. Therefore, burden tests and non-burden tests have been developed, by combining signals of multiple variants in a chromosomal region or a functional unit. This inevitably incorporates some neutral variants into the test statistics, which can dilute the power of statistical methods. To guard against the noise caused by neutral variants, we here propose an ‘adaptive combination of *P*-values method’ (abbreviated as ‘*ADA*’). This method combines per-site *P*-values of variants that are more likely to be causal. Variants with large *P*-values (which are more likely to be neutral variants) are discarded from the combined statistic. In addition to performing extensive simulation studies, we applied these tests to the Genetic Analysis Workshop 17 data sets, where real sequence data were generated according to the 1000 Genomes Project. Compared with some existing methods, *ADA* is more robust to the inclusion of neutral variants. This is a merit especially when dichotomous traits are analyzed. However, there are some limitations for *ADA*. First, it is more computationally intensive. Second, pedigree structures and founders' sequence data are required for the permutation procedure. Third, unrelated controls cannot be included. We here show that, for family-based studies, the application of *ADA* is limited to dichotomous trait analyses with full pedigree information.

## Introduction

Studies in genetic epidemiology are important to uncover the genetic architecture of complex human diseases. The development of next-generation sequencing technologies has allowed for the mapping of all genetic variants across the human genome. With this, we can search for rare causal variants (minor allele frequency (MAF) <1%), which are mostly not genotyped in genome-wide association studies (GWAS) but are related to the etiology of complex diseases. Till now, many statistical methods have been proposed for rare variant association testing. Most of them were designed for population-based studies where unrelated cases and controls were recruited and analyzed [1]–[22].

Despite a variety of statistical methods, there are two concerns in population-based rare variant association studies. First, population stratification may cause false-positive results. This issue was tackled since the era of genome-wide association studies (GWAS). In GWAS where most genotyped variants were common, principal component analysis (PCA) [23] and mixed models [24] were proposed as effective methods to deal with population stratification. However, studies of population stratification are still limited for next-generation sequencing data [25]. Existing methods, such as PCA and mixed models, can fail to correct for rare variant stratification [26]. Second, rare causal variants are difficult to observe in general populations, and therefore statistical methods are usually underpowered [27]. Although burden tests [2]–[5], [27] and non-burden tests [7]–[9] have been proposed to aggregate signals of multiple variants, searching for rare causal variants remains challenging.

Family-based study design will play a key role in identifying rare causal variants, because rare causal variants can be enriched in families with multiple affected subjects [28], [29]. Burden tests (such as the weighted sum approach (*WS*) [3], the cumulative minor allele test (*CMAT*) [22]), and non-burden tests (such as the sequence kernel association test (*SKAT*) [7], [8]) have been extended to family-based designs by incorporating within-family correlation structures into the statistics [30]–[40]. For continuous traits, Chen *et al.* [31] has proposed “*famSKAT*” that can account for members' relationships within families. This method was essentially equivalent to the method proposed by Schifano *et al.* [38] and the adjusted sequence kernel association test (abbreviated as “*ASKAT*”) proposed by Oualkacha *et al.* [40], although Schifano *et al.*'s method was not originally designed for rare variant association testing. Svishcheva *et al.* [39] then developed a fast family-based *SKAT* (abbreviated as “*FFBSKAT*”) that was shown to be the fastest method to perform the kernel-based association tests for continuous traits. Svishcheva *et al.* have shown a pure coincidence of the *P*-values calculated by the *famSKAT*, *ASKAT*, and the *FFBSKAT* software programs [39].

Testing for effects of rare variants individually is known to be underpowered. To strengthen association signals, both the burden tests and the non-burden tests combine information of multiple rare variants in a gene/region. This inevitably incorporates many neutral variants into the test statistics. Adaptive combination of *P*-values method (abbreviated as ‘*ADA*’) has been shown to outperform the burden tests (e.g., *WS* and the variable threshold approach [5]) and the non-burden tests (e.g., *SKAT*) in rare variant association testing for unrelated subjects, because *ADA* is more robust to the inclusion of neutral variants [13], [21]. Taking this advantage, we here extend the *ADA* method to deal with pedigree data, and compare its power performance with that of the burden test [32], the kernel statistic [32], and the *FFBSKAT* method [39] (*famSKAT* [31] and *ASKAT* [40] are essentially equivalent to *FFBSKAT*). We also apply the method to the Genetic Analysis Workshop 17 (GAW 17) data [27], [41]. Some family-based association testing methods were designed for trio data (or trios plus unrelated controls), such as the rare-variant extensions of the transmission disequilibrium test (rvTDT) [36], [42]. Therefore, these methods are not compared here.

## Materials and Methods

Let *Y_i_* be the trait value of the *i*th subject (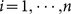). Suppose there are *L* loci in the chromosomal region of interest, and let 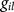 be the genotype score at the *l*th locus of the *i*th subject (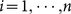, 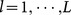). Under the assumption of additive genetic model, 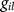 is the number of minor alleles, i.e., 0, 1, or 2. The statistic to test for the association between the trait and the *l*th marker is
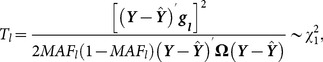
(1)where 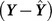 is the vector of residuals after adjusting for covariates (e.g., age, gender), 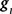 is the genotype score vector at the *l*th marker for the *n* subjects, 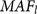 is the MAF of the *l*th marker calculated using founders, and 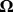 is an *n*×*n* matrix of genetic correlations of these *n* subjects. For autosomes, the (*i*, *j*)th element of 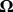 is 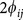, where 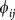 is the kinship coefficient of the *i*th and the *j*th subjects. Because the kinship coefficient of subjects belonging to different pedigrees should be 0, 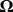 is a block-diagonal matrix with block sizes as the sizes of pedigrees.

The test statistic in Equation (1) has an approximate 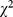 distribution with 1 degree of freedom. This statistic is essentially equivalent to the statistic proposed by Thornton and McPeek [43] (see Equation 1 in [43]). Phenotypes and genotypes are treated as fixed and random, respectively. This retrospective view allows us to correct the ascertainment bias when recruiting pedigrees through affected subjects. After performing *L* tests for the *L* markers, we have *P*-values 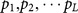. Suppose we consider *J* candidate truncation thresholds, 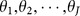. Summarizing the *L* markers, the significance score under the *j*th truncation threshold is 
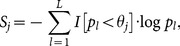
(2)where 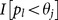 is an indicator variable coded as 1 if the *l*th marker has a *P*-value smaller than 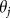 (the *j*th truncation threshold) and 0 otherwise. Throughout this study, the candidate truncation thresholds are specified as 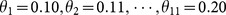, suggested by the *ADA* method for population-based studies [13], [21]. Using a wider range of *P*-value truncation thresholds, say, 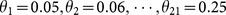, does not contribute a noticeable power gain to *ADA* (results not shown).

**Figure 1:**
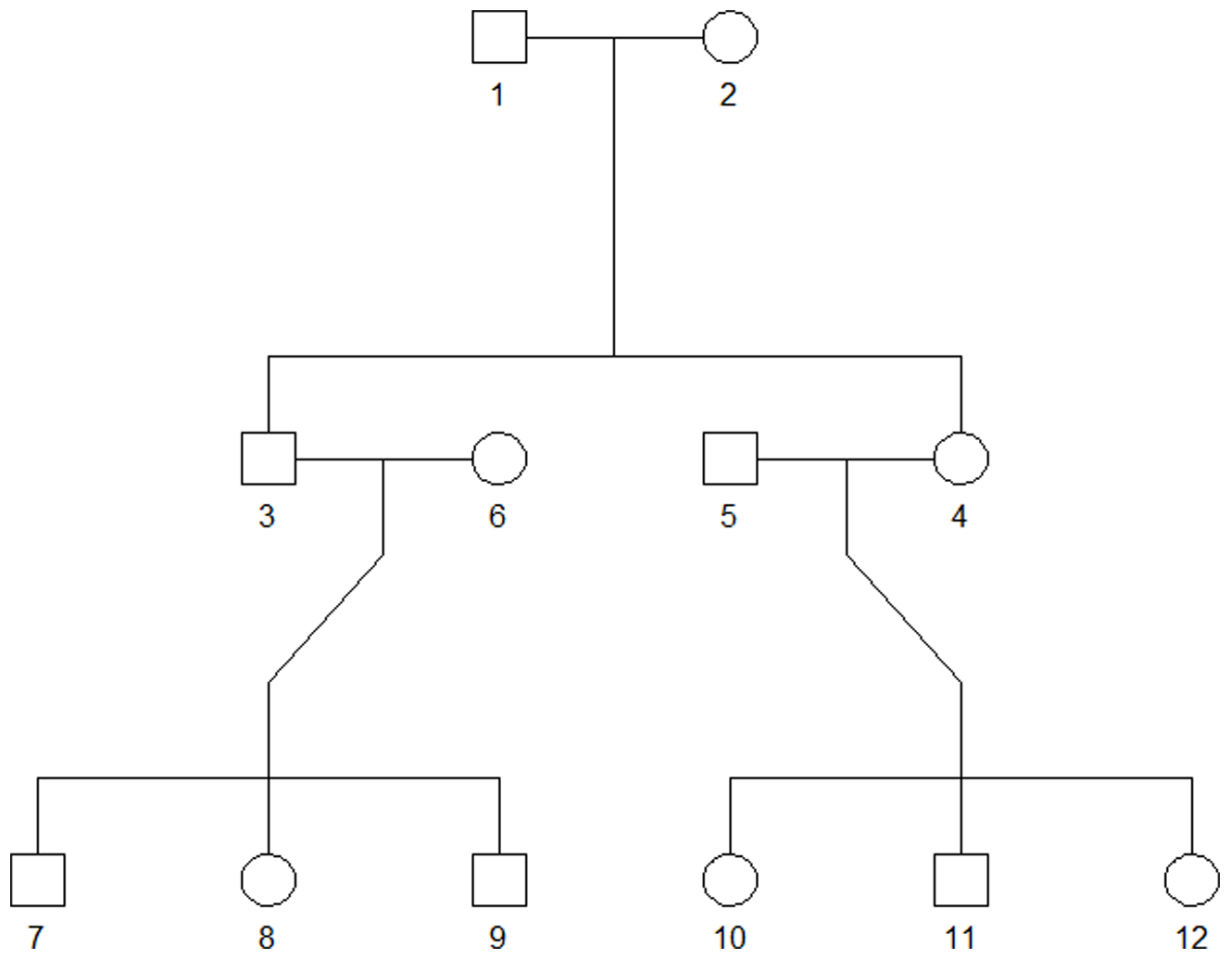
The family structure simulated by SeqSIMLA.

Because multiple *P*-value truncation thresholds are considered, to correct for multiple testing, statistical significance must be obtained with permutations. We first construct the distribution of the significance score *S_j_* under the null hypothesis, where the transmission of haplotypes from parents to offspring is completely random, conditional on parental genotypes [44], [45]. For example, the family shown by Fig. 1 consists of 12 members. Conditional on the genotypes of No. 1 and No. 2, the probability that No. 3 has the observed/unobserved pattern of allelic transmission is 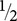 under the null hypothesis. Given the founders' (Nos. 1, 2, 5, 6) genotypes, this family consists of more than 2^8^ = 256 possible patterns of allelic transmission. With a total of *N* families, there are at least 256*^N^* different permutations of the genotype data.

The above permutation procedure was extended from that used for trio data [44], [45]. Unambiguous haplotype phases are not always required in the process. For example, No. 3 has a probability of 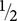 to possess the observed pattern of allelic transmission, and then there is no need to change his genotypes. The probability of owning unobserved pattern of allelic transmission is also 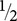. In this situation, No. 3's number of minor alleles at the *l*th locus (0, 1, or 2, representing three different genotype scores) is 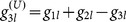, where 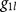 and 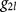 are the genotype scores at the *l*th locus of Nos. 1 and 2, respectively; 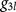 is No. 3's original observed genotype score. Conditional on 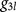 and 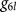 belonging to Nos. 3 and 6, the unobserved pattern of allelic transmission for No. 7 is 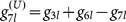. On the other hand, given 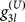 and 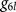, haplotype phases of Nos. 3 and 6 are required to determine the genotype scores of Nos. 7–9. An haplotype-phasing software package (such as Beagle [46]) is used to infer the most-likely haplotype pairs for Nos. 3 and 6. The genotype scores of Nos. 7–9 are then determined by randomly drawing one haplotype from No. 3 and one from No. 6.

Suppose we perform *B* permutations, say, *B* = 1000. For the *b*th permutation, the significance score under the *j*th truncation threshold can be calculated with Eq. (2), denoted as 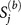. The statistical significance of 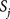 is obtained by comparing it with 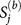, 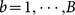. The *P*-value of 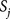 is estimated as 
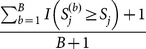
, for each truncation threshold (*j* = 1, …, *J*), where 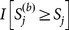 is an indicator variable coded as 1 if 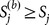 and 0 otherwise. Similarly, the *P*-value of 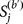 for the 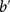
th permutation is 
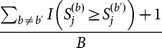
, for *j* = 1, …, *J* and 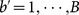. We can then find the minimum *P*-values across the *J* candidate truncation thresholds for the observed sample and the *b*th permuted sample, denoted as *MinP* and *MinP* ^(*b*)^, respectively. The “adjusted *P*-value” is estimated as 
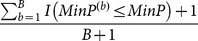
. This method is referred to as “*ADA*”, because the optimal *P*-value truncation threshold is driven adaptively according to the data.

### Simulation Study

We generated sequence data with the SeqSIMLA software [47], which was designed to simulate sequence data for family samples. SeqSIMLA used GENOME [48] as the default sequence generator that could efficiently simulate sequence data according to the standard coalescent model [49]–[52]. In this way, we aim to evaluate statistical methods with simulated data that can reflect realistic DNA sequences. In each simulation, 50, 80 or 100 three-generation families each with 12 members were generated. The family structure was shown by Fig. 1. For each subject, a chromosome region with *m* = 50, 100, or 150 SNPs/SNVs (single-nucleotide polymorphisms/single-nucleotide variants) was simulated. Dichotomous traits and continuous traits were considered respectively.

When evaluating type-I error rates, no causal locus was specified. When evaluating power, five SNPs/SNVs (Nos. 10, 20, 30, 40, 50) were assumed to be causal. We did not restrict all causal variants to be rare/uncommon, because in reality causal variants could also be common. Let ‘signal proportion’ be the fraction of causal variants out of all variants. The signal proportion in our simulations was set at one of the three levels: 0.033 ( = 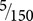), 0.05 ( = 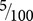), 0.1 ( = 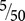).

When dichotomous traits were considered, the overall population attributable risk (PAR) for all causal loci was assumed to be 0.05, 0.10, 0.15, 0.20, 0.25, and 0.30, respectively. Therefore, the marginal PAR for each causal locus was 0.01, 0.02, 0.03, 0.04, 0.05, and 0.06, respectively. In the SeqSIMLA software [47], the genotype relative risk (GRR) of the *j*th causal SNP/SNV was:
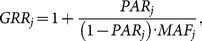
(3)where *PAR_j_* and *MAF_j_* were the PAR and the population MAF of that SNP/SNV, respectively. S1 Fig. showed the distribution of GRRs of causal SNPs/SNVs when the overall PAR for all causal loci was assumed to be 0.05, 0.10, 0.15, 0.20, 0.25, and 0.30, respectively (the marginal PAR for each causal locus was 0.01, 0.02, 0.03, 0.04, 0.05, and 0.06, respectively). A variant with a smaller frequency was assumed to have a larger GRR, following the model in many previous contributions [3], [18]–[20], [47]. For a founder, SeqSIMLA randomly sampled two haplotypes {*H*_1_, *H*_2_} from the population sequence pool created by GENOME [48]. According to the SeqSIMLA software [47], the disease status of this subject was determined by
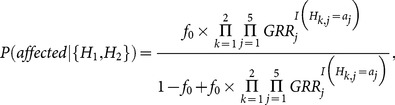
(4)where 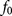 was the baseline penetrance specified as 0.05, 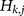 was the allele at the *j*th causal SNP/SNV on the haplotype 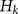 (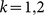), and 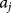 was the minor allele at the *j*th causal SNP/SNV (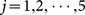) [47]. Given parental haplotypes, a child's haplotypes were formed by randomly selecting one from the father and one from the mother, and the child's disease status was again determined by Eq. (4). Based on this equation, a subject with no causal variant would have a probability of 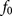 (baseline penetrance) to be diseased, while a subject with more causal variants would have a larger probability to be diseased.

When continuous traits were simulated, five SNPs/SNVs (Nos. 10, 20, 30, 40, 50) were assumed to be quantitative trait loci (QTLs). Causal variants were not restricted to be all rare or uncommon, because in reality common variants can be causal as well. Let 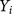 be the trait value of the *i*th subject. It was determined by the model 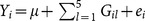, where 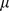 was the overall mean of the trait, 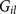 was the genotypic value at the *l*th QTL of the *i*th subject (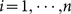, 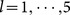) which followed a normal distribution (with a mean of 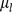, 0, or 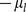 for 2, 1, and 0 minor alleles at the *l*th QTL, respectively [47]), and 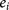 was the error term for the *i*th subject following a normal distribution as well. According to the default setting of the SeqSIMLA software [47], the genetic effects of QTLs were all assumed to be additive, and 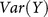 and 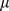 were both specified at 100. These two values were not critical because SeqSIMLA software [47] actually controlled the proportion of 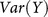 explained by each QTL (denoted as 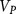). The value of 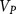 was assumed to be 0.001, 0.002, 0.003, 0.004, 0.005, and 0.006 for each QTL, respectively. The corresponding proportion of variance explained by all the five QTLs was therefore 0.005, 0.01, 0.015, 0.02, 0.025, or 0.03.

### Tests under Comparison

We compared *ADA* with the broad classes of the burden test (referred to as “*Burden*”) [32], the kernel test for family data (referred to as “*Kernel*”) [32], and the *FFBSKAT* method [39]. *Burden* and *Kernel* were implemented with the R package “pedgene” [32]; *FFBSKAT* was performed with the package “FFBSKAT” (http://mga.bionet.nsc.ru/soft/FFBSKAT/) [39]. *FFBSKAT* was performed only when continuous traits were considered, because it could not analyze dichotomous traits. Following the default setting of *FFBSKAT* [39] and *Kernel* [32], the (*j*, *j*)th element of the diagonal weighting matrix 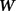 was set as 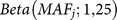, where 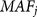 was the MAF of the *j*th genetic variant. The *P*-values of *ADA* were obtained with 1,000 permutations.

## Results

### Type-I Error Rates

By setting the PAR or the proportion of variance explained by causal SNPs at exactly 0%, we evaluated type-I error rates by performing 10,000 replications. In each replication, 50 three-generation families each with 12 members (shown in Fig. 1) were generated. For each subject, a chromosome region containing 100 SNPs/SNVs was simulated. Table 1 shows that all the tests (three for dichotomous traits and four for continuous traits) are valid in the sense that their type-I error rates match the nominal significance levels.

**Table 1:** Type-I error rates based on 10,000 replications.

When dichotomous traits were considered					
nominal significance level	0.01	0.02	0.03	0.04	0.05
*ADA* a	0.0099	0.0201	0.0298	0.0399	0.0503
*Kernel*	0.0097	0.0197	0.0284	0.0375	0.0474
*Burden*	0.0107	0.0210	0.0311	0.0403	0.0501

When continuous traits were considered					
*ADA* a	0.0105	0.0203	0.0301	0.0403	0.0502
*Kernel*	0.0085	0.0180	0.0282	0.0383	0.0484
*Burden*	0.0103	0.0201	0.0303	0.0402	0.0498
*FFBSKAT*	0.0105	0.0181	0.0278	0.0359	0.0464

a *P*-values were estimated based on 1,000 permutations.

### Power Comparisons

When we evaluated power, 1000 replications were performed under each scenario. In total, there were 54,000 replications in power evaluation for dichotomous traits and continuous traits, respectively (three levels of *m* (50, 100, or 150) × three levels of family numbers (50, 80, or 100) × six levels of PAR (0.05, 0.1, …, 0.3) or proportion of variance explained by causal SNPs (0.005, 0.01, …, 0.03) ×1000 replications for each scenario). Across all these 108,000 replications, there were totally 540,000 ( = 108,000×5) causal SNPs/SNVs (because 5 causal SNPs/SNVs or QTLs were specified in each replication). Among these 540,000 causal variants, approximately 45% were rare (MAF<1%), ∼62% were uncommon/rare (MAF<5%), and ∼ 38% were common (MAF≥5%). In our simulation setting, causal variants were not limited to be rare, because in real situations causal variants could also be common.

**Figure 2:**
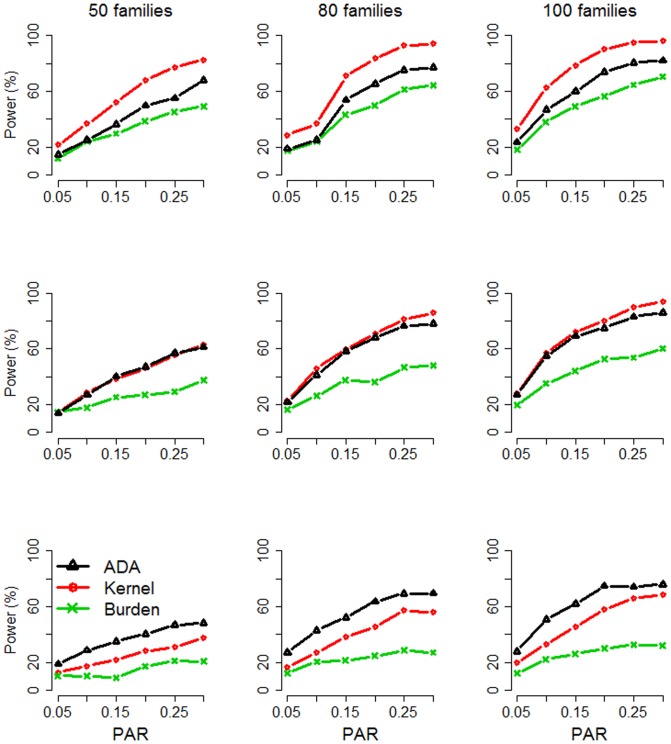
Power Comparison for dichotomous traits. The figure shows the empirical power given the significance level of 0.05. Top row: 50 variants included in the tests; middle row: 100 variants; bottom row: 150 variants. The *x*-axis is the overall population attributable risk (PAR) for all causal loci, whereas the *y*-axis is the power.

**Figure 3:**
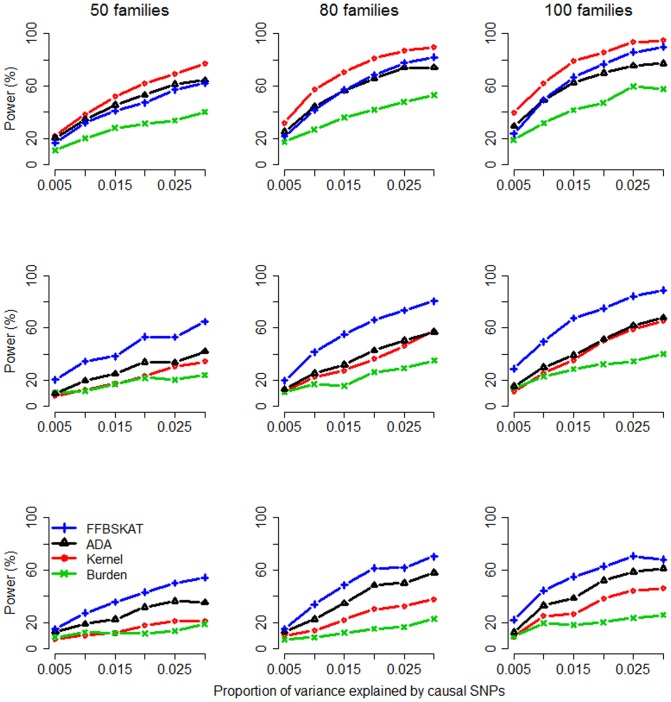
Power Comparison for continuous traits. The figure shows the empirical power given the significance level of 0.05. Top row: 50 variants included in the tests; middle row: 100 variants; bottom row: 150 variants. The *x*-axis is the proportion of variance explained by causal SNPs, whereas the *y*-axis is the power.

Figs. 2 and 3 present the power for dichotomous traits and continuous traits, respectively. When dichotomous traits were studied (Fig. 2), *Kernel* was the most powerful method when *m* = 50 (signal proportion  = 0.1), whereas *ADA* had the best performance when *m* = 150 (signal proportion  = 0.033). When *m* = 100 (signal proportion  = 0.05), these two methods had comparable performance. *Burden* was the uniformly least powerful test among the methods we compared, regardless of the size of *m* (50, 100, or 150). To conclude, when the signal proportion was larger, *Kernel* was more powerful; when the signal proportion was smaller, *ADA* took the advantage of truncating noise variants and therefore was more powerful.

When continuous traits were studied (Fig. 3), *Kernel* was again the most powerful method when *m* = 50 (signal proportion  = 0.1). When the signal proportion was getting lower and lower (or, *m* was getting larger and larger), *Kernel* had a more substantial power loss. *Burden* was again the least powerful method, regardless of the size of *m* (50, 100, or 150). Compared with *Kernel*, *FFBSKAT* and *ADA* were less vulnerable to the inclusion of neutral variants. *FFBSKAT* became the most powerful method when *m* = 100 (signal proportion  = 0.05) or when *m* = 150 (signal proportion  = 0.033).

### Application to Genetic Analysis Workshop 17 Simulated Data

We then applied the four tests to the Genetic Analysis Workshop 17 (GAW 17) simulated data [41]. The GAW 17 data set was designed to mimic a subset of data that might be generated in a full exome investigation for a complex disease. To reflect realistic human genomes, real sequence data were generated based on the 1000 Genomes Project [53]. The data set consisted of 697 subjects from eight large pedigrees, in which 202 founders had genotypes randomly selected from the 1000 Genomes Project. The MAFs ranged from 0.07% to 16.5%. These founders included 66 Tuscan, 50 Luhya, 28 Japanese, 19 Han Chinese, 18 Denver Chinese, 12 CEPH (European-descent residents of Utah), and 9 Yoruban samples. Pedigrees included four generations, and relatives were as distant as second cousins. The causal SNPs/SNVs were listed in Table 2. Phenotype simulations were performed multiple times to generate 200 replications. With this simulated data set, we could evaluate the power performance of the four statistical methods, given a more general pedigree structure and a more realistic sequence composition [41]. The *β* column was the change in mean quantitative trait due to a copy of minor allele.

**Table 2:** Analysis of the Genetic Analysis Workshop 17 simulated data.

Causal gene	No. of SNPs/SNVs	No. of causal SNPs/SNVs	Signal proportion	Causal SNP/SNV	MAF	*β*	Power to detect the causal gene^a^ (significance level = 0.05)			
							*FFBSKAT*	*ADA* b	*Kernel*	*Burden*
*ARNT*	18	5	0.28	C1S6533	0.011478	0.589734	0.175	0.065	0.005	0
				C1S6537	0.000717	0.642689				
				C1S6540	0.001435	0.323662				
				C1S6542	0.002152	0.488219				
				C1S6561	0.000717	0.625721				
*ELAVL4*	10	2	0.20	C1S3181	0.000717	0.795093	0.19	0.07	0	0.005
				C1S3182	0.000717	0.328748				
*FLT1*	35	11	0.31	C13S320	0.001435	0.18047	1	0.76	0.335	0.38
				C13S399	0.000717	0.457361				
				C13S431	0.017217	0.732566				
				C13S479	0.000717	0.839669				
				C13S505	0.000717	0.38582				
				C13S514	0.000717	0.549816				
				C13S522	0.027977	0.623466				
				C13S523	0.066714	0.653351				
				C13S524	0.004304	0.596704				
				C13S547	0.000717	0.549214				
				C13S567	0.000717	0.090586				
*FLT4*	10	2	0.20	C5S5133	0.001435	0.120761	0.74	0.39	0.03	0.065
				C5S5156	0.000717	0.385374				
*HIF1A*	8	4	0.50	C14S1718	0.000717	0.251622	0.03	0.015	0	0
				C14S1729	0.002152	0.329088				
				C14S1734	0.012195	0.220448				
				C14S1736	0.000717	0.228202				
*HIF3A*	21	3	0.14	C19S4799	0.000717	0.174668	0.175	0.035	0.01	0.005
				C19S4815	0.000717	0.51468				
				C19S4831	0.000717	0.265181				
*KDR*	16	10	0.63	C4S1861	0.002152	0.598271	0.925	0.845	0.72	0.87
				C4S1873	0.000717	0.715613				
				C4S1874	0.000717	0.503025				
				C4S1877	0.000717	1.17194				
				C4S1878	0.164993	0.149975				
				C4S1879	0.000717	0.610938				
				C4S1884	0.020803	0.318125				
				C4S1887	0.000717	0.312058				
				C4S1889	0.000717	1.17194				
				C4S1890	0.002152	0.417977				
*VEGFA*	6	1	0.17	C6S2981	0.002152	1.13045	1	1	1	0.995

a Power to detect a causal gene  =  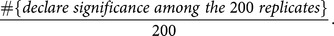

b *P*-values were estimated based on 1,000 permutations.

To analyze this data set, we first obtained residuals (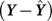 in Eq. (1)) by regressing the trait values on age and smoking status. Table 2 listed the results by *FFBSKAT*, *ADA*, *Kernel*, and *Burden*. The *P*-values of *ADA* were estimated based on 1,000 permutations. Based on the power to detect causal genes, these methods were roughly ranked as *FFBSKAT*> *ADA*> *Kernel* ≈ *Burden*. *FFBSKAT* was the most powerful method. It was based on the linear mixed effects model, in which the kinship relatedness was captured by the random effects terms (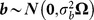, as described in our Introduction section). In the GAW 17 data set, the eight pedigrees were all quite large (see S2 Fig.). The linear mixed effects model (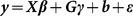) partitions the total phenotypic variance into several parts. When pedigrees are larger, this model is a better choice because 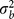 can be estimated more accurately. (We used SeqSIMLA2 [54] to simulate 1,380 subjects based on two pedigree structures: (A) 20 pedigrees each with 69 members (Fig. 1 of [54]), and (B) 115 pedigrees each with 12 members (Fig. 1). The variance of the polygenic effects, 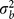, was specified at 45 in SeqSIMLA2 [54]. With 1,000 replications, the mean of 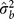 estimated by the *lmekin* function [55] was 44.33 and 47.51 for cases (A) and (B), respectively.) Therefore, *FFBSKAT* was more powerful than other methods when analyzing the GAW 17 large pedigree data.

## Discussion

Many statistical methods were proposed for rare variant association testing, but most of them were designed for population-based studies. Among the family-based rare variant association testing methods, some extend the transmission disequilibrium test [56], [57] and focus on parent-child trio data [36], [37]. Some methods are eligible for analyzing pedigree data (including but not limited to trios), and they can be categorized as the burden tests and the non-burden tests (e.g., *famSKAT* [31], [38], *FFBSKAT* [39], *Kernel* [32]). The non-burden tests were shown to be more powerful than the burden tests in most situations [32], [33].

Among the non-burden tests compared in this work, *FFBSKAT* [39] (and *famSKAT* [31], [38]) can only deal with continuous traits, but it is more powerful than the other methods (*ADA*, *Kernel*, *Burden*) when the pedigree is larger (see the GAW 17 data analysis) or when the signal proportion is smaller (see the bottom two rows of Fig. 3).

In population-based studies, *ADA* is robust to the inclusion of neutral variants, and therefore it has been shown to outperform the burden tests and the non-burden tests (e.g., *SKAT*) [13], [21]. In this work, we extend *ADA* to family-based studies and compare it with *Kernel* and *Burden*, the two commonly used methods for dichotomous traits. Simulation studies show that *ADA* is more powerful than other two competitors when the percentage of causal variants is smaller (see the bottom row of Fig. 2). On the contrary, *Kernel* is more powerful when the percentage of causal variants is larger (the top row of Fig. 2). *Burden* has the least power across the simulation scenarios we have investigated. The comparison between *Kernel* and *Burden* is consistent with that found by previous studies [32], [33].

*Kernel*, *Burden*, and *FFBSKAT* can provide analytical *P*-values when the sample size is large. *ADA* searches for the optimal threshold among multiple *P*-value truncation thresholds. Therefore, permutation is required to assess the statistical significance, and so *ADA* needs more computational time than other methods. To be more computationally efficient, *ADA* can be combined with a sequential Monte Carlo algorithm [58]. For simulated data sets containing 50 families and 50 SNPs/SNVs, *ADA* on average needs ∼168.2 sec, *Kernel* or *Burden* takes ∼37.6 sec, and *FFBSKAT* needs ∼4.3 sec. This was measured on a Linux platform with an Intel Xeon E5-2690 2.9 GHz processor and 2 GB memory. Although the computation time of other competitors is much shorter than that of *ADA*, *ADA* is more robust to the inclusion of neutral variants when dichotomous traits are studied (see Fig. 2). But when continuous traits are analyzed, *FFBSKAT/Kernel* outperforms *ADA* when the signal proportion is smaller/larger (see Fig. 3).

Rare causal variants may play an important role in the etiology of complex diseases [59]–[64], but they are challenging to detect through single-locus tests [1], [2], [65], [66]. Combining variants' signals in a chromosomal region and testing for association with a grouping statistic is a commonly used strategy. Compared with the burden test (*Burden*) and the non-burden test (*Kernel*), *ADA* is more robust to the inclusion of neutral variants when dichotomous traits are analyzed. However, there are some limitations for *ADA*. First, because this method is more computationally intensive, it is not realistic to apply it to genome-wide sequencing data. Second, pedigree structures and founders' sequence data are required for the permutation procedure implemented in *ADA*. Third, unrelated controls cannot be included in the *ADA* analyses. This work shows that, for family-based studies, the application of *ADA* is limited to dichotomous trait analyses with full pedigree information.

## Supporting Information

S1 Fig **The distribution of genotype relative risk (GRR) of causal SNPs/SNVs when the overall population attributable risk (PAR) for all causal loci was assumed to be 0.05, 0.10, 0.15, 0.20, 0.25, and 0.30, respectively.** Therefore, the marginal PAR for each causal SNP/SNV was 0.01, 0.02, 0.03, 0.04, 0.05, and 0.06, respectively.
(PDF)

S2 Fig **Structures of the eight pedigrees (accordingly, from pedigree 1, 2, …, 8) in the Genetic Analysis Workshop 17 data set, plotted by the R package “kinship2”.**
(PDF)
