# Supplementary material for: Adaptive Combination of P-Values for Family-Based Association Testing with Sequence Data
Source: PLoS One. 2014 Dec 26;9(12):e115971. doi: 10.1371/journal.pone.0115971 (PMC4277421; doi:10.1371/journal.pone.0115971)
Supplement: S1 Fig — The distribution of genotype relative risk (GRR) of causal SNPs/SNVs when the overall population attributable risk (PAR) for all causal loci was assumed to be 0.05, 0.10, 0.15, 0.20, 0.25, and 0.30, respectively. Therefore, the marginal PAR for each causal SNP/SNV was 0.01, 0.02, 0.03, 0.04, 0.05, and 0.06, respectively. (PDF) [file pone.0115971.s001.pdf]

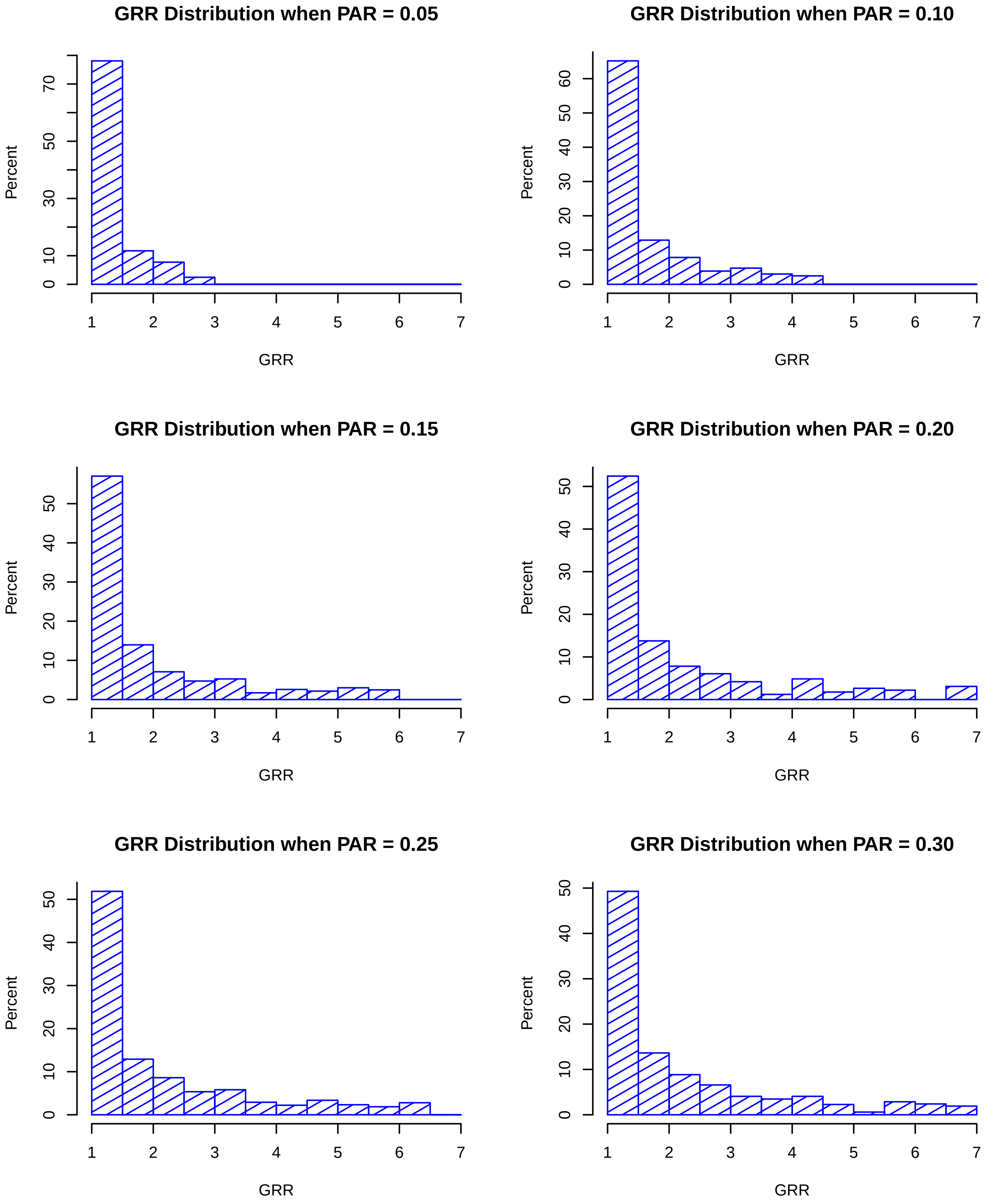
GRR Distribution when PAR = 0.05
Percent
0
10
30
50
70
1
2
3
4
5
6
7
GRR
GRR Distribution when PAR = 0.10
Percent
0
10
20
30
40
50
60
1
2
3
4
5
6
7
GRR
GRR Distribution when PAR = 0.15
Percent
0
10
20
30
40
50
1
2
3
4
5
6
7
GRR
GRR Distribution when PAR = 0.20
Percent
0
10
20
30
40
50
1
2
3
4
5
6
7
GRR
GRR Distribution when PAR = 0.25
Percent
0
10
20
30
40
50
1
2
3
4
5
6
7
GRR
GRR Distribution when PAR = 0.30
Percent
0
10
20
30
40
50
1
2
3
4
5
6
7
GRR
