# Supplementary figures and images for: Adaptive Combination of P-Values for Family-Based Association Testing with Sequence Data
Source: PLoS One. 2014 Dec 26;9(12):e115971. doi: 10.1371/journal.pone.0115971 (PMC4277421; doi:10.1371/journal.pone.0115971)

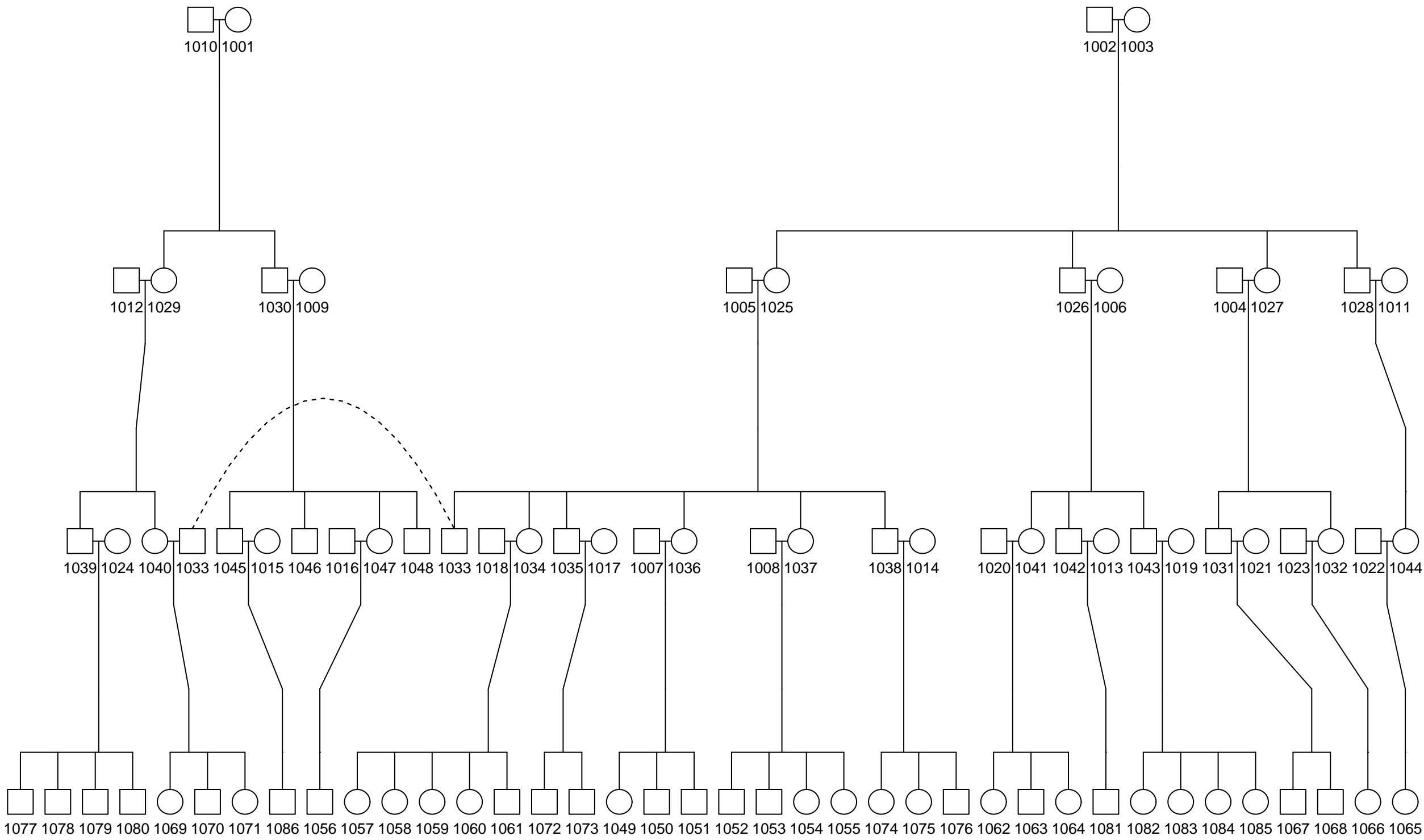

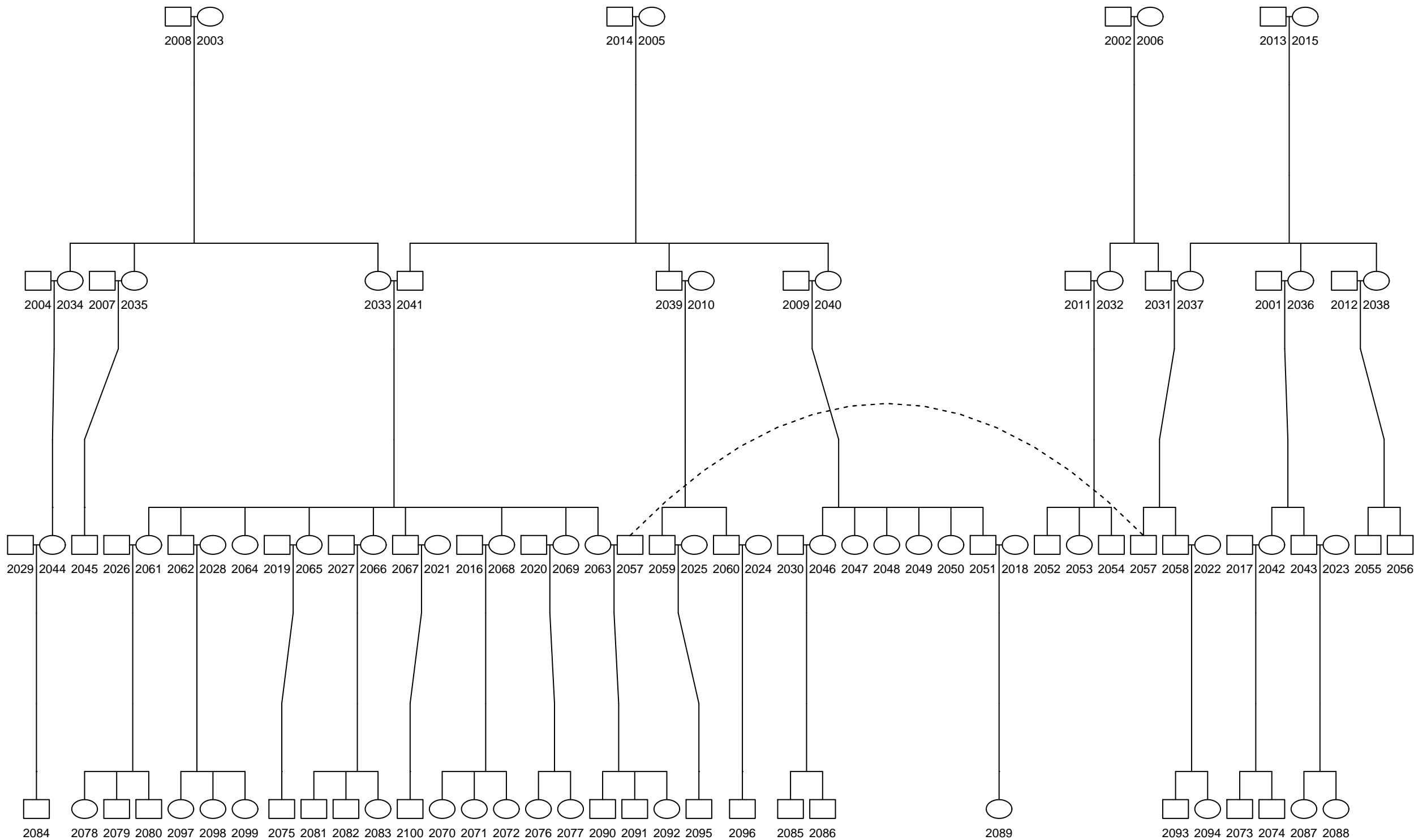

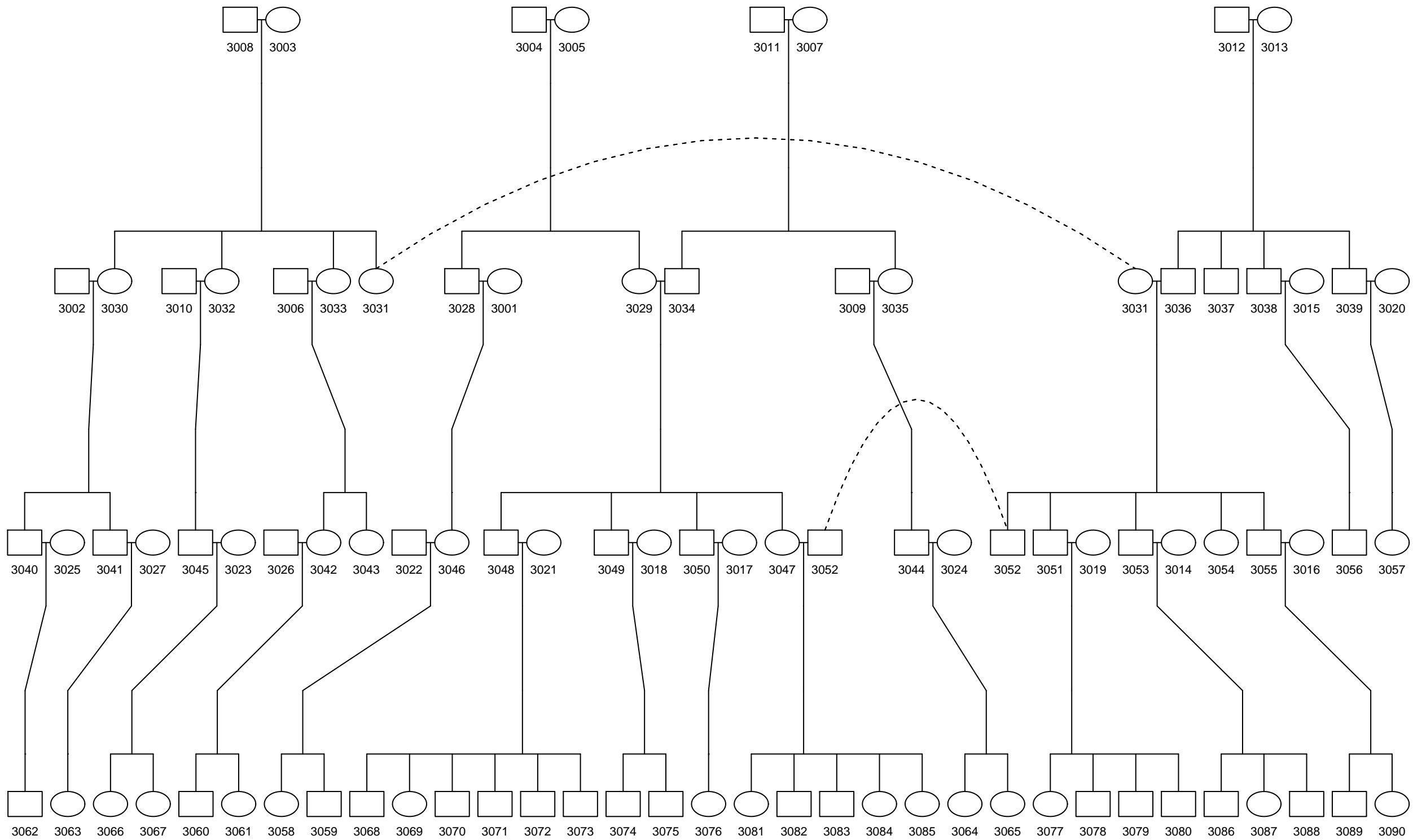

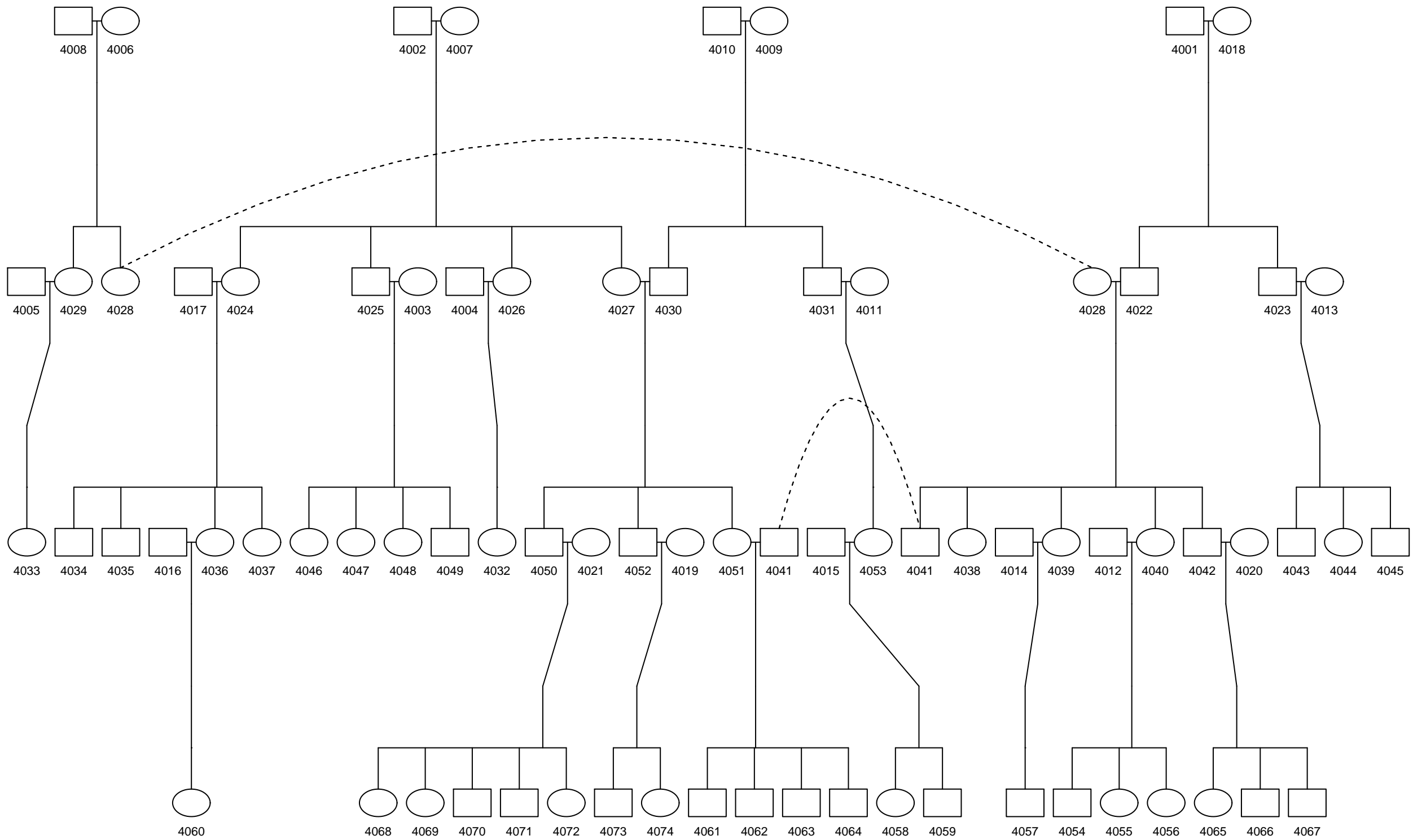

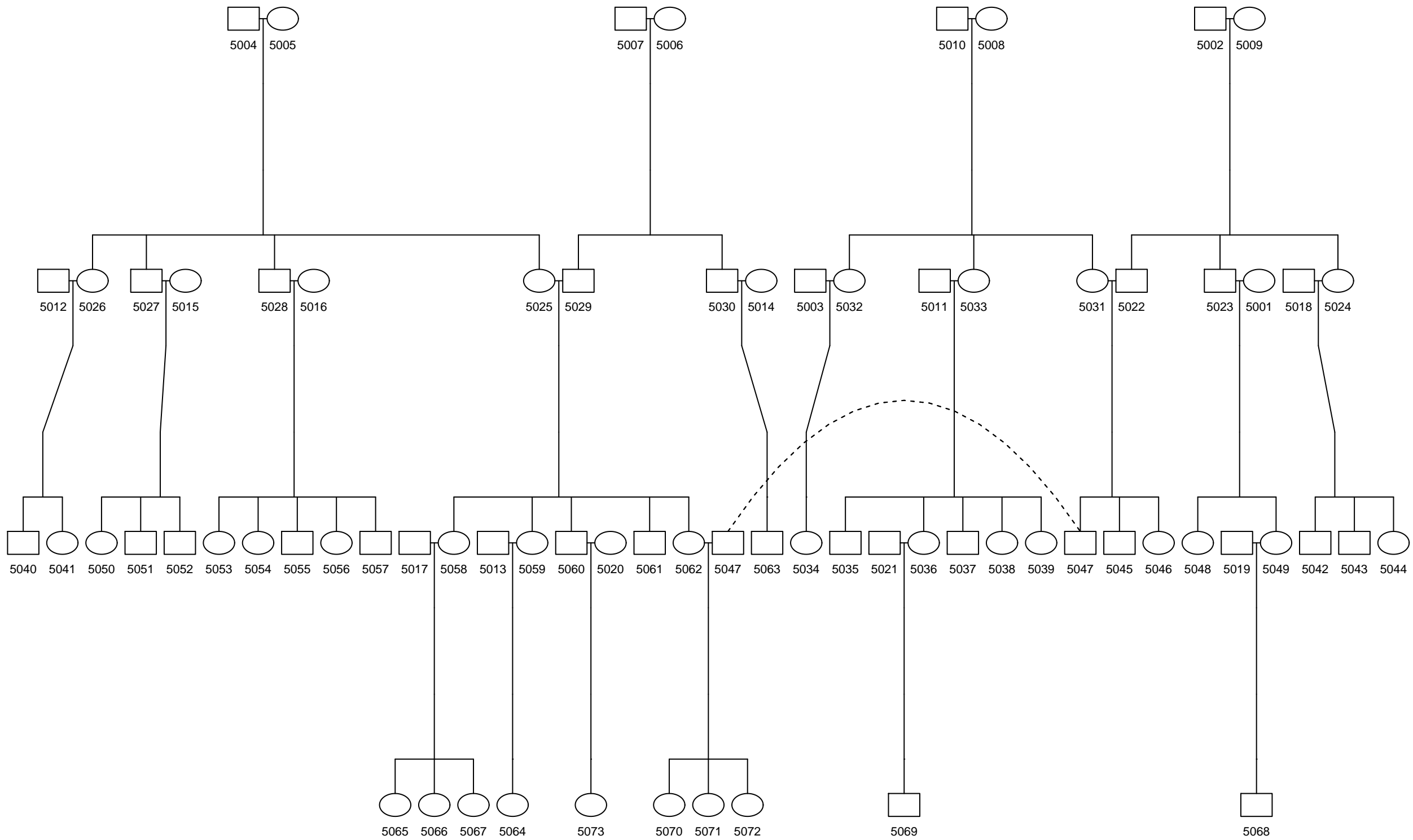

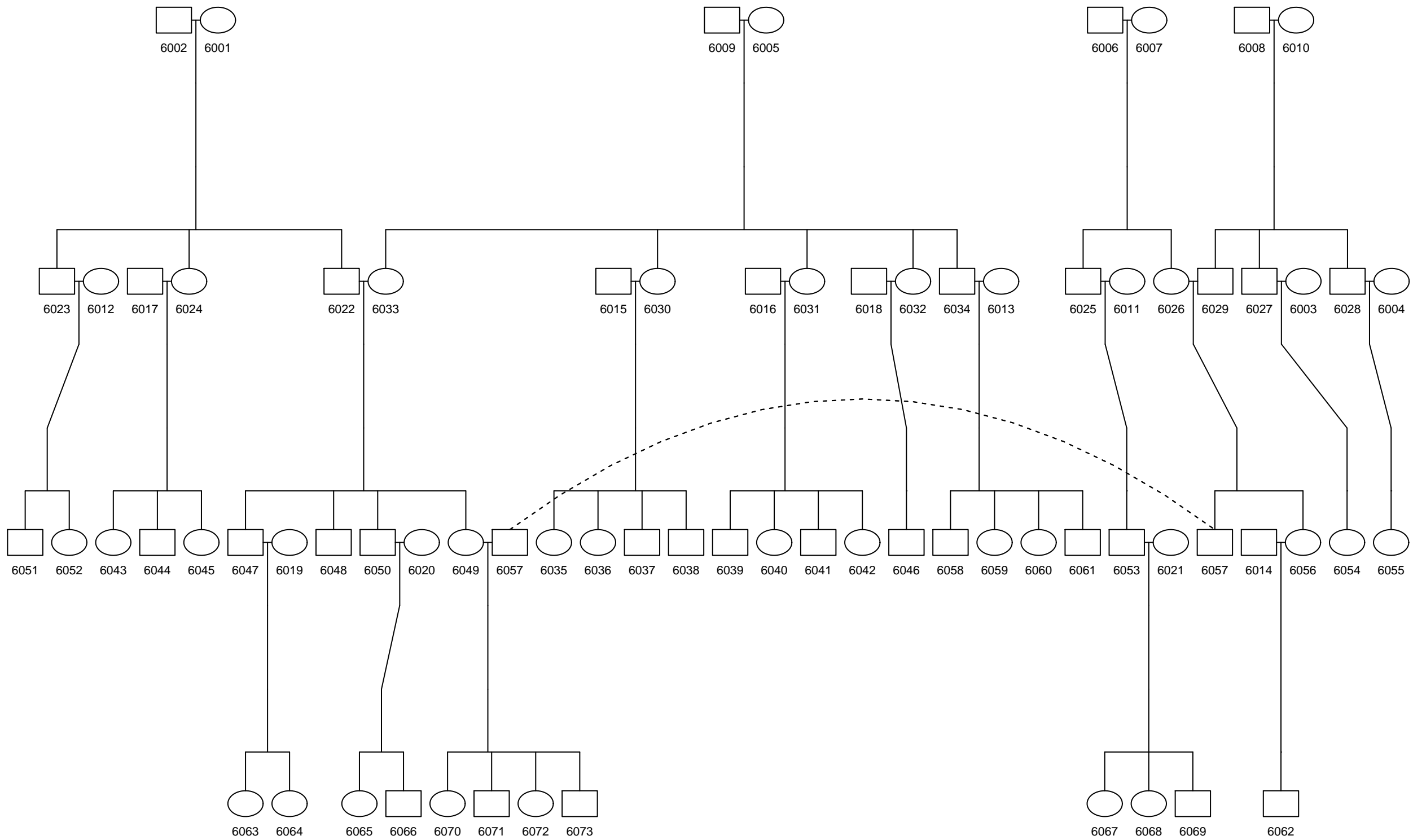

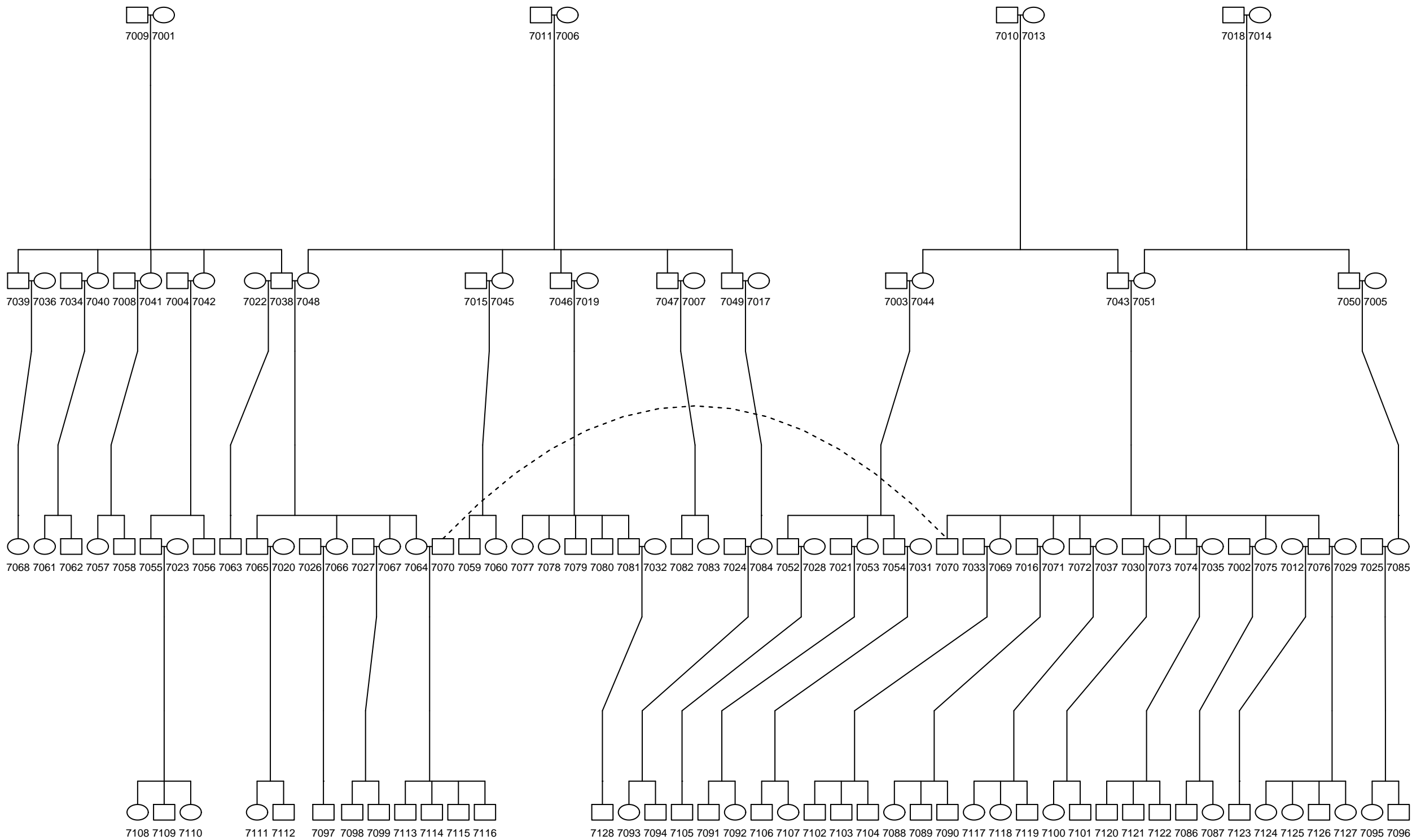

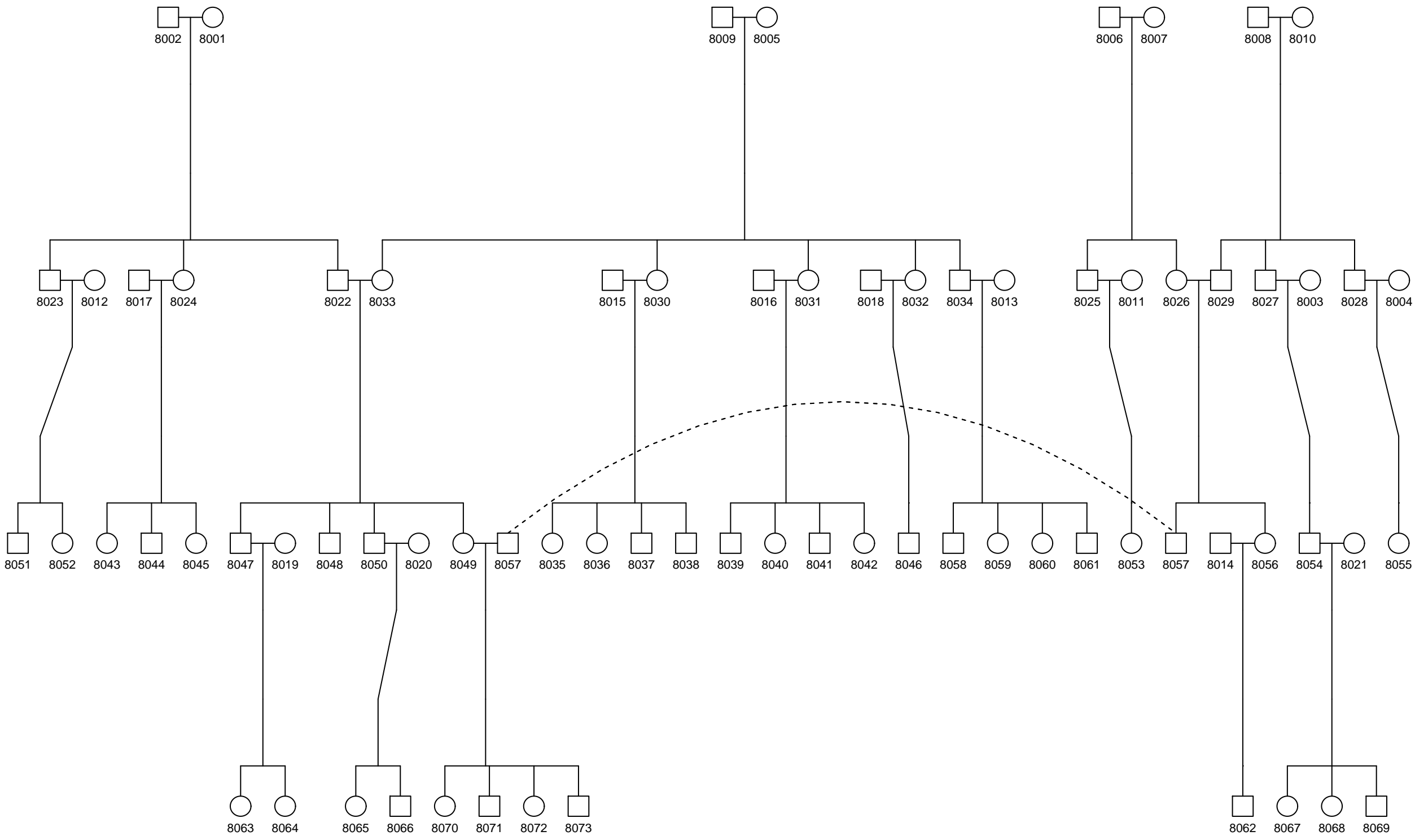

Supplement: S2 Fig — Structures of the eight pedigrees (accordingly, from pedigree 1, 2, …, 8) in the Genetic Analysis Workshop 17 data set, plotted by the R package “kinship2”. (PDF) [file pone.0115971.s002.pdf]
